# Identification of an oxidative stress-related prognostic signature, tumor immune microenvironment, and development of subtypes for breast cancer patients receiving neoadjuvant chemotherapy

**DOI:** 10.1097/MD.0000000000046343

**Published:** 2026-05-12

**Authors:** Zhongmei Shi, Xiaoli Shen, Zhiyun Mao, Xiuping Hang

**Affiliations:** a Department of Laboratory Medicine, Dongtai Hospital of Traditional Chinese Medicine, Dongtai City, Jiangsu Province, China; b Department of Laboratory Medicine, The People’s Hospital of Rugao, Rugao, Jiangsu Province, China.

**Keywords:** breast cancer, immune infiltration, neoadjuvant chemotherapy, oxidative stress, prognosis

## Abstract

The formation of tumors and the reaction to anticancer treatments are significantly influenced by the regulation of oxidative stress. Nevertheless, it is still unknown how useful oxidative stress-related genes are for forecasting how well a treatment will work and the prognosis of breast cancer. OSRG features were built using least absolute shrinkage and selection operator regression algorithm. The prognostic performance was evaluated via Kaplan–Meier analysis, receiver operating characteristic curves, and multivariate Cox regression in trainingand validation cohorts. Quantitative reverse transcriptase polymerase chain reaction (qRT-PCR) was used to detect the expression level of oxidative stress-related genes in the features. Furthermore, we investigated the correlation between OSRG features and molecular subtypes, immunological landscape, chemotherapeutic response, and clinicopathological features. A robust 7-gene risk model was established and validated. The high-risk group’s relapse-free survival was shorter, and the receiver operating characteristic curve’s area under the curve values at 1, 3, and 5 years were satisfactory, according to internal and external validation. Furthermore the 2 groups differed significantly in their sensitivity to chemotherapeutic drugs and immunological microenvironment. Based on OSRG expression, patients were stratified into 4 molecular clusters with divergent relapse-free survival and immune activity. OSRG signatures and molecular clusters serve as potent predictors of the immune microenvironment, therapeutic response, and survival prognosis, potentially providing new perspectives into the management and tailored therapy of breast cancer patients.

## 1. Introduction

Breast cancer (BC) has become a main global health threat and a leading cause of cancer incidence among women worldwide.^[1,2]^ In clinical practice, there are various treatment options for BC including surgery, endocrine chemotherapy, radiotherapy, and hormonal therapy, depending on the tumor subtype, histological classification, grade, and stage.^[3]^

Patients with locally advanced cancers frequently get neoadjuvant chemotherapy (NAC) to shrink the tumor and improve surgical success rates.^[4,5]^ Because of its cosmetic advantages and lower risk of several surgical problems, NAC can help patients with non-advanced BC as well.^[6,7]^ The intervention of NAC brings new hope to improve the outcomes and the quality of life of BC patients, and provides a novel strategy for the comprehensive treatment of BC.^[8]^

The imbalance between the generation of reactive oxygen species (ROS) and antioxidant systems in cells in reaction to damaging stimuli is known as oxidative stress (OS). The development of cancer and the reaction to anticancer treatment are significantly influenced by the regulation of intracellular OS. Cell homeostasis is maintained under normal physiological settings by the equilibrium between ROS generation and antioxidants.^[9]^ Numerous tumorigenesis-related signaling pathways are involved in regulating ROS production directly or indirectly by affecting the metabolic mechanisms of cancer cells.^[10]^ The supplementation of oxidative supplements during conventional treatment for BC patients is beneficial to improve the therapeutic effect.^[11]^ To fully comprehend how oxidative stress contributes to the development of cancer and how treatment response affects it, more research is necessary.^[12]^

Novel biomarker development and the identification of important therapeutic targets open up new avenues for cancer patient prevention, prognosis, and early diagnosis.^[13]^ In this study, we developed an OSRG prognostic signature to internally and externally predict relapse-free survival (RFS), and explored the relationship between immune infiltration status, drug sensitivity and clinicopathological characteristics in BC patients receiving NAC to guide clinical practice. Additionally, this signature is a useful predictor that can help determine whether immunotherapy is beneficial for BC patients.

## 2. Methods

### 2.1. Data acquisition and processing

RNA-seq data queue for breast invasive carcinoma from the Cancer Genome Atlas (the cancer genome atlas, https://portal.gdc.cancer.gov/) database for FPKM format download. Moreover, RNA-Seq and clinical data of BC patients receiving NAC were obtained by extracting the GSE25066 cohort from the Gene Expression Omnibus (GEO) as a training cohort, and GSE16446 and GSE22226 as test cohorts. To look for oxidative stress-related genes (OSRGs), the GeneCards database was filtered. Additional analysis was carried out on the OSRGs that were deemed eligible based on correlation scores >20.

### 2.2. Construction of predictive features based on prognostic OSRGs expression

The GSE25066 cohort’s prognostic OSRGs was determined using univariate Cox proportional hazard regression analysis, with a cutoff value of *P* <.01. The optimal model was then identified using least absolute shrinkage and selection operator (LASSO) in conjunction with ten cross-validation screenings, with the use of the R package “glment.” Genes with nonzero regression coefficients were selected for subsequent multivariate Cox regression analysis. The following was the LASSO regression model: riskScore = Σ coef (genei) × exp (genei) (coef: model gene coefficient, exp: expression level of the model gene;)

### 2.3. External validation of prognostic features

First, the “surv” function in the “Surv” package is used to conduct KM survival analysis in the GSE25066 datasets. Using the “coxph” function in the “survival” package, univariate and multivariate Cox regression analysis were conducted between gene expression and clinical characteristics to demonstrate that riskScore is an independent predictor of RFS in BC patients.

In addition, survival receiver operating characteristic (ROC) curves were plotted using the “timeROC” package, and area under the curve values for 1-year, 3-year, and 5-year survival were calculated to assess the sensitivity and specificity of the prognostic model. The model’s prediction performance was then confirmed using the same method on the GSE16446 and GSE22226 datasets.

### 2.4. Cell culture

Human normal mammary epithelial cells MCF-10A and human BC cell lines MCF-7, T47D, SK-BR-3, MDA-MB-231, and BT-549 were acquired from the Chinese Academy of Sciences Cell Bank (CBCAS, Shanghai, China). MDA-MB-231, BT-549 and SK-BR-3 cells were cultured in Dulbecco Modified Eagle medium and MCF-7 was cultured in RPMI 1640 medium, MCF-10A cells were cultured in DMEM/F12 medium supplemented with 20 ng/μL epidermic growth factor. All media were supplemented with 10% FBS (HyClone, Logan) and 2 antibiotics (penicillin and streptomycin). Following digestion with 0.25% trypsin (Invitrogen), all cell lines were cultured in a cell incubator (Forma 3110, Thermofisher) set at 37 °C with 5% CO2.

### 2.5. Quantitative real-time polymerase chain reaction

Following the manufacturer’s recommendations, total RNA was extracted from cellines using the TRIzol® reagent (Takara Bio, Inc.). One microgram of total RNA was reverse-transcribed using the Fast All-in-One RT kit to produce cDNA. Gene expression patterns for MAOB, CYCS, XBP1, and GAPDH were assessed by qRT-PCR with Roche Light Cycler 480 QPCR equipment (Roche Diagnostics, Germany) and a SYBR Green PCR Master Mix (Applied TaKaRa, Otsu, Shiga, Japan). The 2-ΔΔCT method was used to process the relative expression levels, and they were then normalized to the internal housekeeping gene GAPDH. Every experiment was carried out 3 times. The primers listed below were utilized:

CYCS-forward: CTTTGGGCGGAAGACAGGTC;

CYCS-reverse: TTATTGGCGGCTGTGTAAGAG;

XBP1-forward: CCCTCCAGAACATCTCCCCAT;

XBP1-reverse: ACATGACTGGGTCCAAGTTGT;

MAOB-forward: GGAGCTAGGATTGGAGACCTAC;

MAOB-reverse: CCCTGAAGGGGTATGATTTGC;

GAPDH-forward: AGAAAAACCTGCCAAATATGATGAC;

GAPDH-reverse: TGGGTGTCGCTGTTGAAGTC.

### 2.6. Evaluation of chemotherapy drug sensitivity

By using the “oncoPredict” package to compute the maximum half inhibition Concentration index (IC50) using information from the Cancer Drug Sensitivity Genomics Database (GDSC) based on the gene expression profiles provided in the 3 datasets, the response of patients in the 2 risk groups to different medications was examined.

### 2.7. Description of immune infiltrating landscape

The CIBERSORT platform was used to evaluate the proportion of 22 immune cells in BC patients. Based on the expression levels of genes linked to immune cells, the “estimate” R package was used to compute the differences in 3 tumor microenvironment (TME) scores: stromal score (SS), estimate score (ES), and immune score (IS). we also employed Spearman correlation analysis to ascertain the relationship between immune cell demeanor and riskScore. Moreover, a single sample gene set enrichment analysis (ssGSEA) algorithm was used to evaluate the variations in immune function across the 4 subgroups.

### 2.8. Consensus cluster analysis for identification of BC subtypes

ConsensusClusterPlus software package in R was used for consensus clustering based on Euclidean distance and Ward linkage to perform hierarchical clustering, dividing tumor samples into 4 specific molecular clusters based on OSRG expression levels.^[14]^

## 3. Statistical analysis

Analyze the data using R.V.4.1.3. For continuous variables conforming to normal distribution, the Student *t*-test is used for comparison. The non-normally-distributed continuous and categorical variables were compared using the Wilcoxon and chi-square tests, respectively. The difference in RFS between various risk groups and molecular clusters was ascertained using the logarithmic rank test. For all statistical results, the threshold of statistical significance was set as *P <.05; **P <.01; ***P <.001.

## 4. Results

### 4.1. Construction of the oxidative stress-related signature in BC

**Figure 1.**
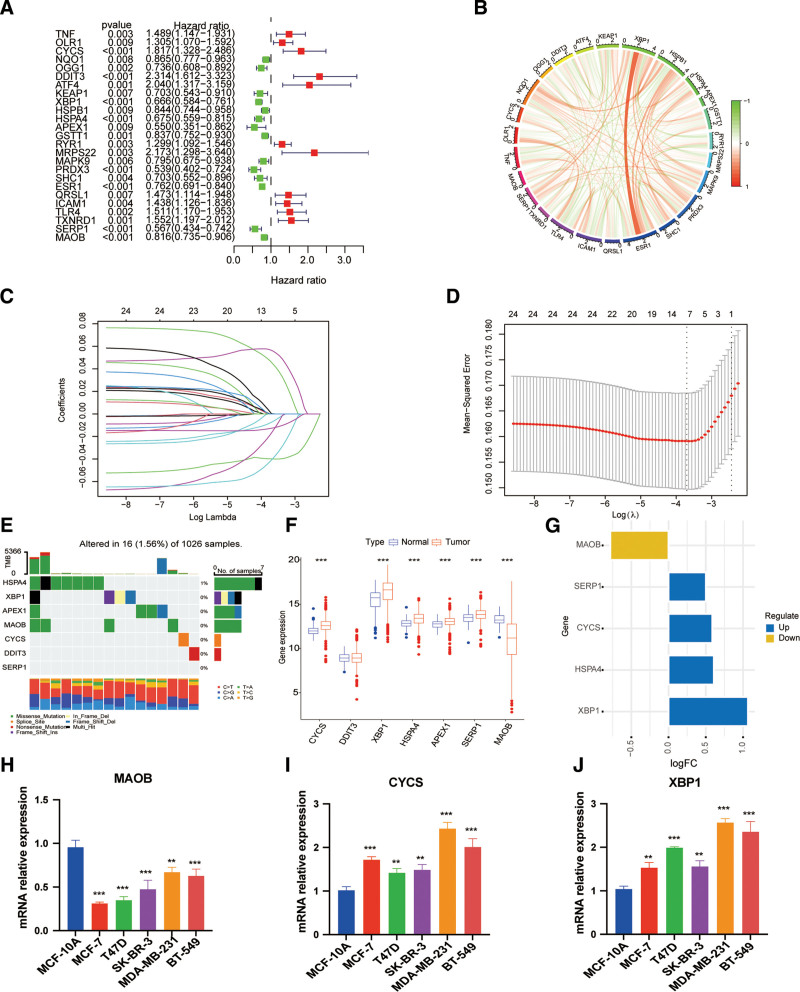
(A) The univariable Cox HR regression was employed to determine the HR and *P*-value of specific OSRGs (criteria: *P*-value <.01). (B) The 25 OSRGs’ expression interactions in BC. The relationships between OSRGs are depicted by the lines linking them; a positive correlation is shown by red, and a negative correlation is shown by green. (C and D) Seven OSRGs related to BC were identified by Lasso-Cox analysis.(E) 16 of 1026 (1.56%) BC patients experienced 7 OSRG genetic alterations.(F) Expression leves of the 7 OSRGs in normal tissues and BC tissues. (G) The value of logFC of 7 OSRGs. (H–J) qRT-PCR results showed the expression value of the 3 OSRGs in the normal breast and 5 breast cancer cell lines. BC = breast cancer, HR = hazard ratio, OSRGs = oxidative stress-related genes, qRT-PCR = quantitative reverse transcriptase polymerase chain reaction.

The oxidative stress-related signature was constructed using the GSE25066 dataset, comprising 507 samples, as the training cohort. For external validation, the GSE16446 dataset with 107 samples and the GSE22226 dataset with 129 samples were utilized as test cohorts. First, we performed a univariate Cox regression analysis in GSE25066 datasets. Among 195 OSRGs in (Table S1, Supplemental Digital Content, https://links.lww.com/MD/Q855) retrieved from the GeneCards database, 25 OSRGs were identified to be connected to RFS, With a *P*-value of <.01 (Fig. 1A). To discover interactions among the 25 OSRGs, we used Pearson correlation analysis to reveal correlations between these genes (Fig. 1B). The prognostic potential of the 25 OSRGs was further refined using LASSO-penalized Cox regression to prevent overfitting and select the most predictive features. This analysis identified an optimal lambda value, at which only 7 genes, including MAOB, CYCS, XBP1, HSPA4, DDIT3,APEX1, and SERP1, retained nonzero coefficients,we finally identified 7 OSRGs for building the prediction model (Fig. 1C and D).

### 4.2. Gene mutation and expression landscape of model genes

Among 1026 samples, only 16 samples had mutations, with a mutation rate of 1.56%. We found that only HSPA4 was mutated, and the other genes did not show any mutations in the BC samples (Fig. 1E). Then, in contrast to normal breast tissue, breast tumor tissue had considerably lower MAOB expression levels and higher levels of CYCS, XBP1, HSPA4, APEX1, and SERP1 (Fig. 1F). Among them, XBP1 was regarded as a significantly up-regulated gene, while MAOB were regarded as significantly down-regulated genes (Fig. 1G). Results of qRT-PCR in BC cell lines strongly supported our conclusion (Fig. 1H–J). In short, OSRGs present significant heterogeneity in the genetic variation and transcriptome-altering landscape of BC patients, and they might be crucial in controlling the disease’s onset, course, and outcome.

### 4.3. External evaluation of prognostic features associated with oxidative stress

**Figure 2.**
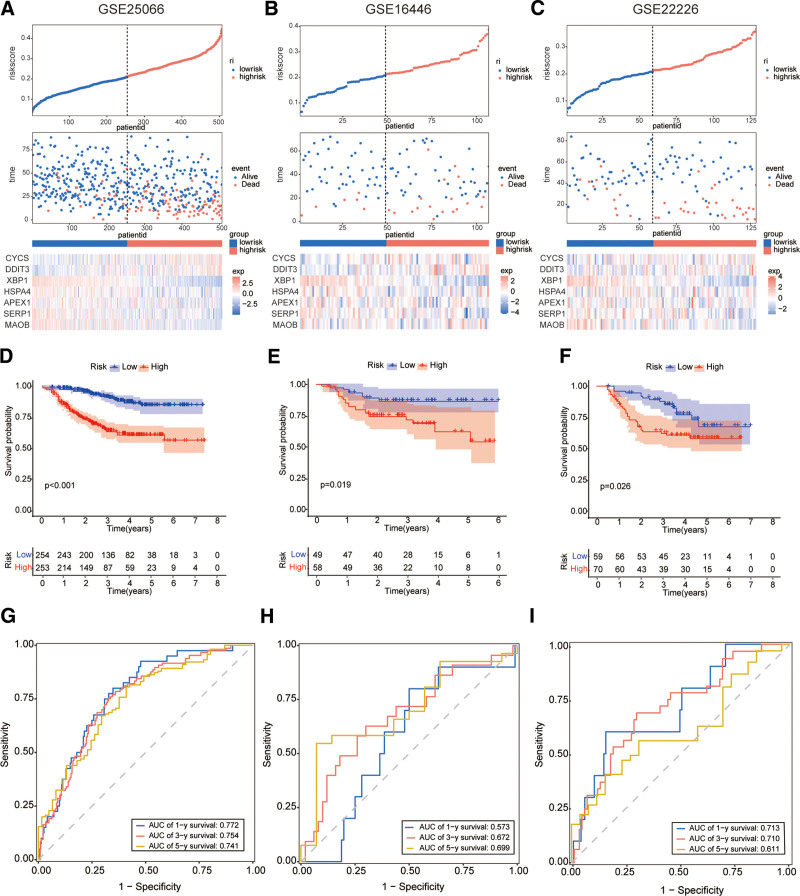
RiskScore can effectively predict the prognosis of BC patients. Distribution of riskScore, scatter plot of vital status by riskScore, and heatmap of the 7 OSRGs’ expression in the high-risk group and the low-risk group in GSE25066 cohort (A), GSE16446 cohort (B), and GSE22226cohort (C). Kaplan–Meier curves for the high- and low-risk patients in GSE25066 cohort (D), GSE16446 cohort (E), and GSE22226cohort (F). Time-dependent ROC curves for GSE25066 (G), GSE16446 (H), and GSE22226 (I) that predict 1-, 3-, and 5-year RFS. BC = breast cancer, RFS = relapse-free survival, ROC = receiver operating characteristic.

**Figure 3.**
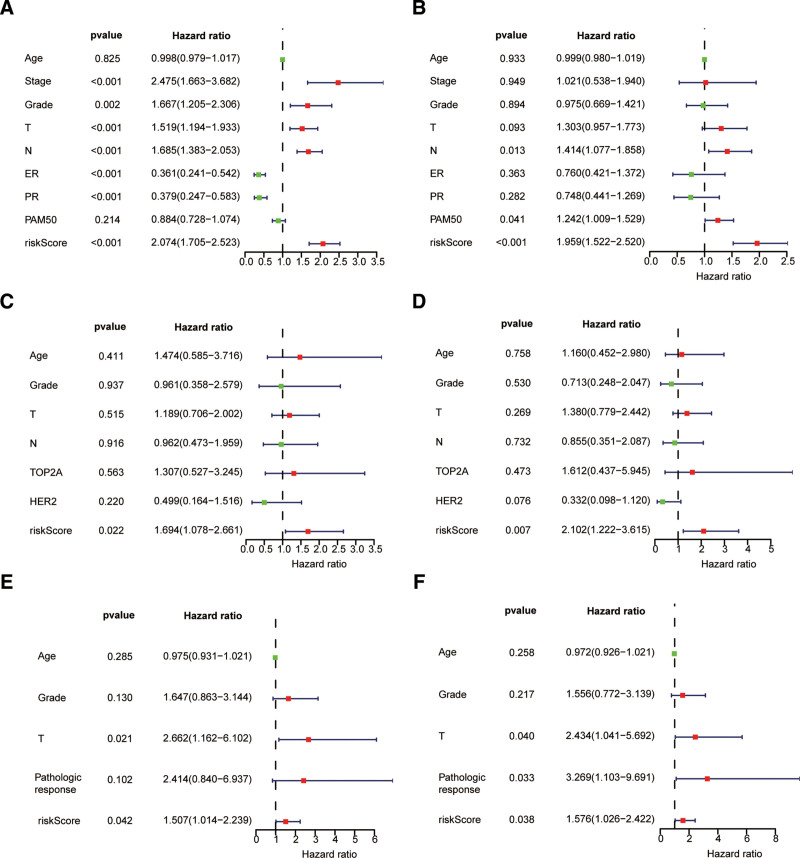
RiskScore can be used as an independent risk factor for the prognosis of BC. Univariate and multivariate Cox regression analyses of riskScore in GSE25066 cohort (A and B), GSE16446 cohort (C and D), GSE22226cohort (E and F). BC = breast cancer.

After calculating the each patient’s riskScore according to the risk model, we used the standard median score calculation of the GSE25066 training cohort to divide the patients with GSE25066, GSE16446 and GSE22226 into groups with high and low risks. The riskScore distribution, survival status, and expression status of each sample in the 3 datasets are shown in (Fig. 2A–C), respectively. The prognosis for the high-risk group was poorer than that of the low-risk group in the GSE25066 datasets (Fig. 2D). According to Figure 2G, the AUC values at 1, 3, and 5 years were 0.772, 0.754, and 0.741, respectively. Next, the predictive performance of prognostic features was tested using the GSE16446 and GSE22226 datasets. Similar survival outcomes were found in the GSE16446 and GSE22226 datasets (Fig. 2E and F). As shown in Figure 2H, the GSE16446 dataset’s 1-, 3-, and 5-year AUC values were 0.573, 0.672, and 0.699, respectively. In GSE22226 datasets, the 1-year, 3-year and 5-year AUC values of were 0.713, 0.710 and 0.611 respectively (Fig. 2I). In the GSE25066 (Fig. 3A and B), GSE16446 (Fig. 3C and D), and GSE22226 (Fig. 3E and F) datasets, univariate and multivariate analyses were conducted to investigate whether characteristics might be utilized as potential factors affecting the prognosis of patients with BC. The results showed that riskScore was found to be an independent risk factor that BC patients were affected by.

### 4.4. Risk scores associated with clinicopathological parameters

**Figure 4.**
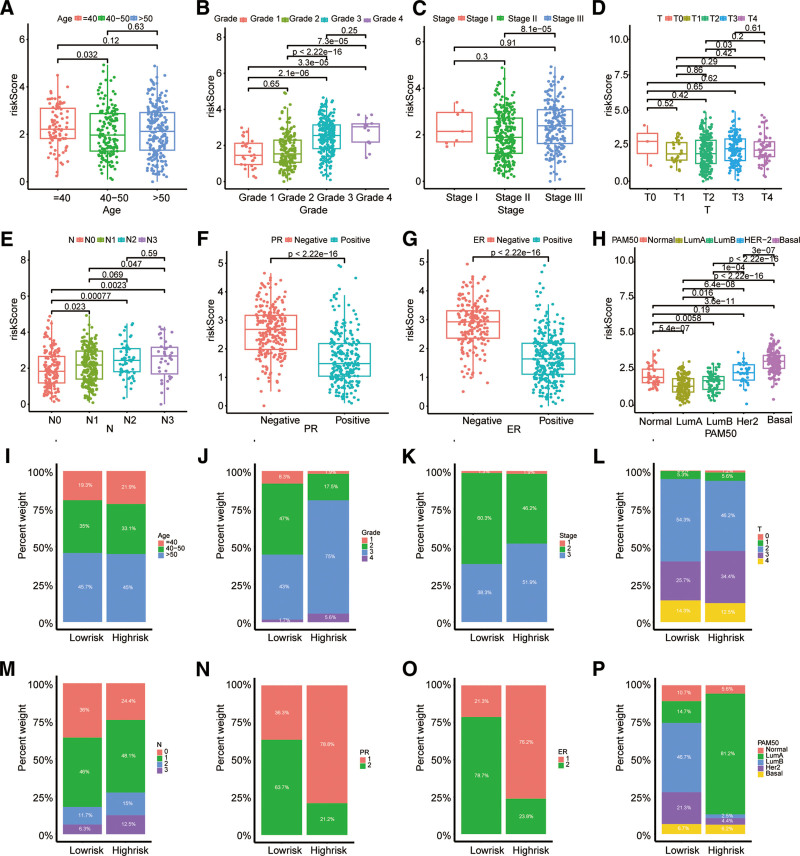
The clinicopathological characteristics of BC are connected with the riskScore. Age (A), grade (B), stage (C), T (D), N (E), PR status (F), ER status (G), PAM50 subtypes(H). (I–P) distributions and percentages of age, grade, stage, T, ER status, PR status and PAM50 subtypes in the 2 groups. BC = breast cancer.

The specific analysis results of box plot and scatter plot showed that riskScore was positively correlated with Grade, T stage, N stage and American Joint Committee on Cancer stage (Fig. 4B–E). In addition, we noted that BC patients with younger age and progesterone receptor and estrogen receptor-negative status appeared to have a higher riskScore (Fig. 4A, F, and G), suggesting a higher recurrence rate. We also saw that HER-2 positive BC indicated a higher riskScore, whereas Luminal BC indicated a lower riskScore (Fig. 4H). Additionally, the distribution and percentage differences of several clinical and pathological characteristics between the high-risk and low-risk groups are displayed using box plots (Fig. 4I–P).

### 4.5. Drug sensitivity evaluation in high and low-risk groups

**Figure 5.**
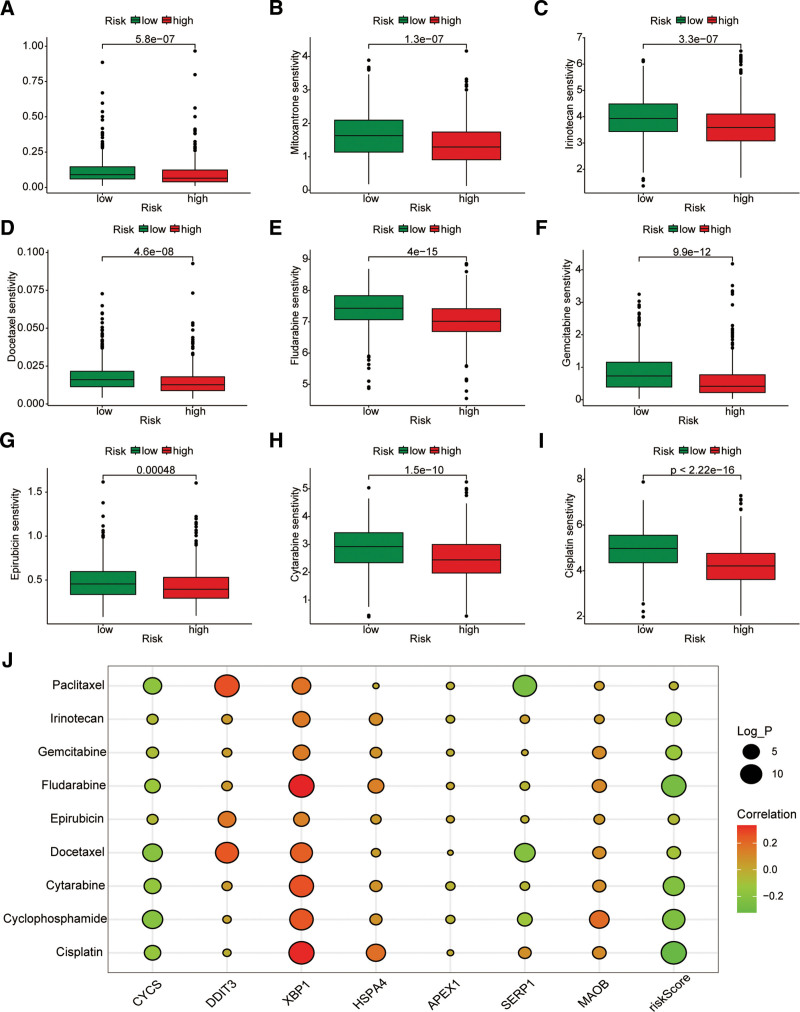
Correlation between OSRG signature and chemotherapy drug sensitivity. Box plots for estimated IC50 of drugs in the different risk groups. Paclitaxel (A), Mitoxantrone (B), Irinotecan (C), Docetaxel (D), Fludarabine (E), Gemcitabine (F), Epirubicin (G), Cytarabine (H), Cisplatin (I). (J) Correlation between the expression levels of 7 genes in the OSRG-related risk model and drug sensitivity. OSRGs = oxidative stress-related genes.

We also investigated if OSRG characteristics may predict the sensitivity of high- and low-risk groups to a number of chemotherapeutic drugs frequently used in the clinical treatment of BC in order to increase the effectiveness of neoadjuvant chemotherapy in BC patients. The IC50 values of chemotherapy drugs, including paclitaxel, mitoxantrone, irinotecan, docetaxel, fludarabine, gemcitabine, epirubicin, cisplatin, and cytarabine, were assessed using the GDSC database. Patients in the high-risk group had considerably lower IC50 levels for these medications, suggesting that they were more susceptible to them (Fig. 5A–I). In addition, we used Spearman correlation coefficient to assess drug responsiveness based on the 7 OSRG expression levels (Fig. 5J).

### 4.6. Comprehensive analysis of oxidative stress gene signatures and immune microenvironment

**Figure 6.**
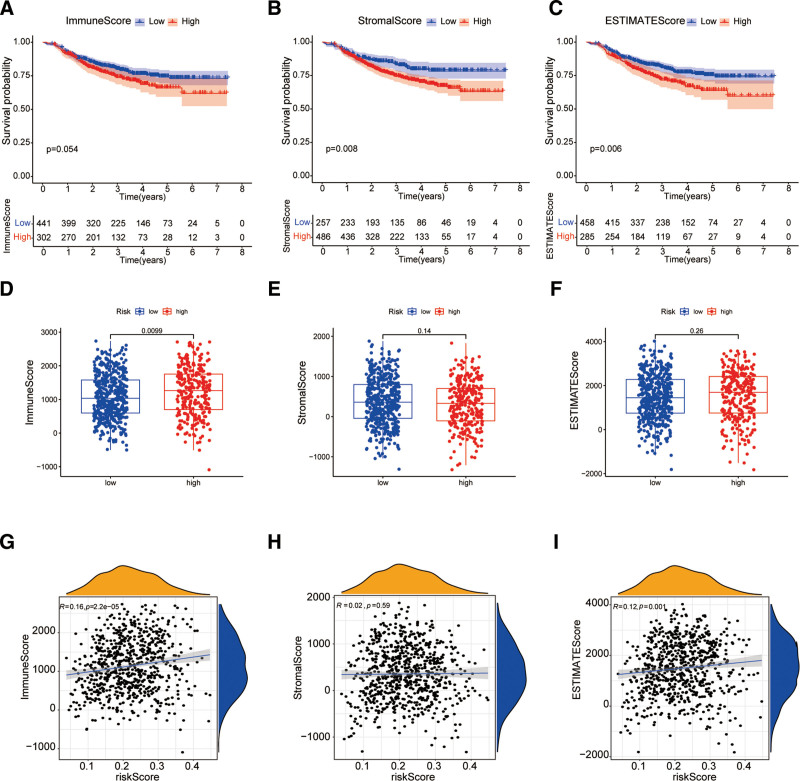
ESTIMATE algorithm was utilized to examine analyze the high-risk and low-risk groups. (A–C) KM curves for the high and low IS, SS, and ES group patients. The box plot (D–F) and scatter plot (G–I) show the association between riskScore and SS, IS, and ES. ES = estimate score, IS = immune score, SS = stromal score.

To investigate the relation between immune landscape of BC patients and riskScore, we first used the ESTIMATE algorithm to evaluate patients’ IS, SS and ES respectively. After calculating the optimal cutoff value, patients were split into 2 groups: those with high IS/SS/ES and those with low IS/SS/ES. Patients with low IS/SS/ES had a considerably better RFS than those with high IS/SS/ES, according to the KM survival analysis (Fig. 6A–C). Then, we further explore the relationship between IS/SS/ES and riskScore. Although there was no significant difference in SS/ES between the 2 groups, the results of Wilcoxon rank sum test showed that the high-risk group had higher IS than low-risk group (Fig. 6D–F). Further, the results of Pearson correlation analysis indicated a positive association between riskScore and both IS (*R* = 0.16, Fig. 6G) and ES (*R* = 0.12, Fig. 6I). However, riskScore was not significantly associated with SS (Fig. 6H).

**Figure 7.**
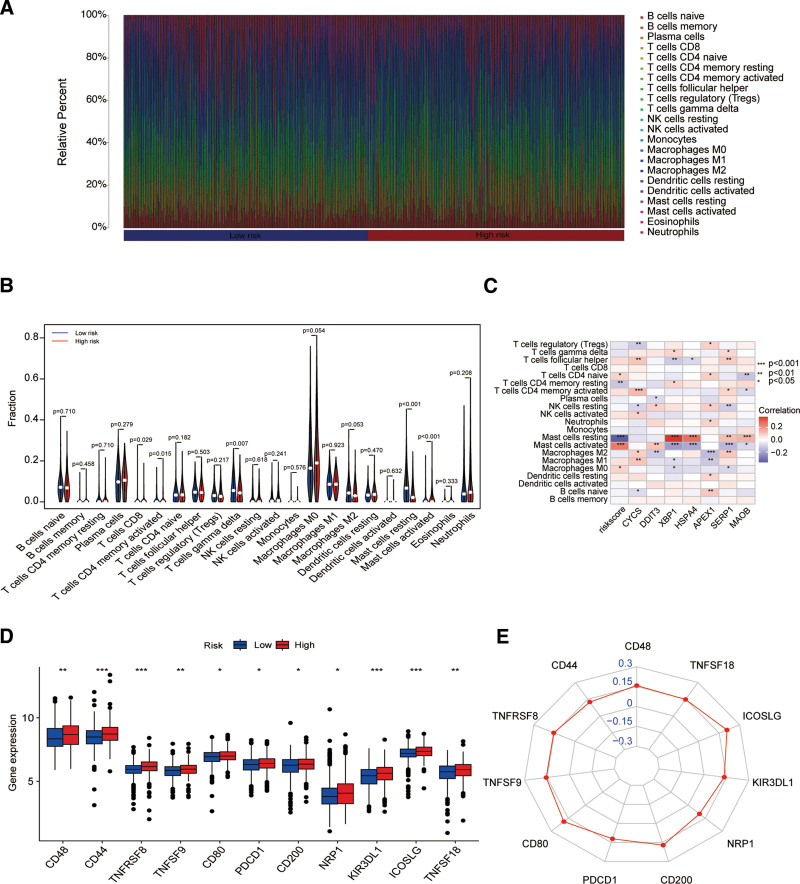
Immune cell infiltration is related to to the OSRG signature. (A) The proportion of 22 immune cell types in each sample. (B) Various immune cell types in low-risk and high-risk groups. (C) Associations of immune cells with riskScore and 7 model genes. (D) Comparisons of immune checkpoint expression levels between 2 distinct groups. (E) Radar charts were used to visualize the correlation of riskScore with immune tests. OSRGs = oxidative stress-related genes.

Subsequently, we utilized the CIBERSORT algorithm to determine the makeup of 22 types of tumor-infiltrating immune cells in patients at low and high-risk groups (Fig. 7A). In comparison to the low-risk group, the high-risk group had higher percentages of T cells CD8, T cells CD4 memory activated, and Mast cells activated, which were positively correlated. The ratio of T cells gamma delta to Mast cells resting was low, showing a negative correlation (Fig. 7B and C). We also examined the potential impact of riskScore on immune checkpoint. It is worth noting that the high-risk group had higher immune checkpoint expression levels than the low-risk group (Fig. 7D and E). This suggests that the immune process appears to be more active in the high-risk group, which offers guidance for therapeutic immunotherapy.

### 4.7. Identification of 4 oxidative stress-related consensus cluster subtypes

**Figure 8.**
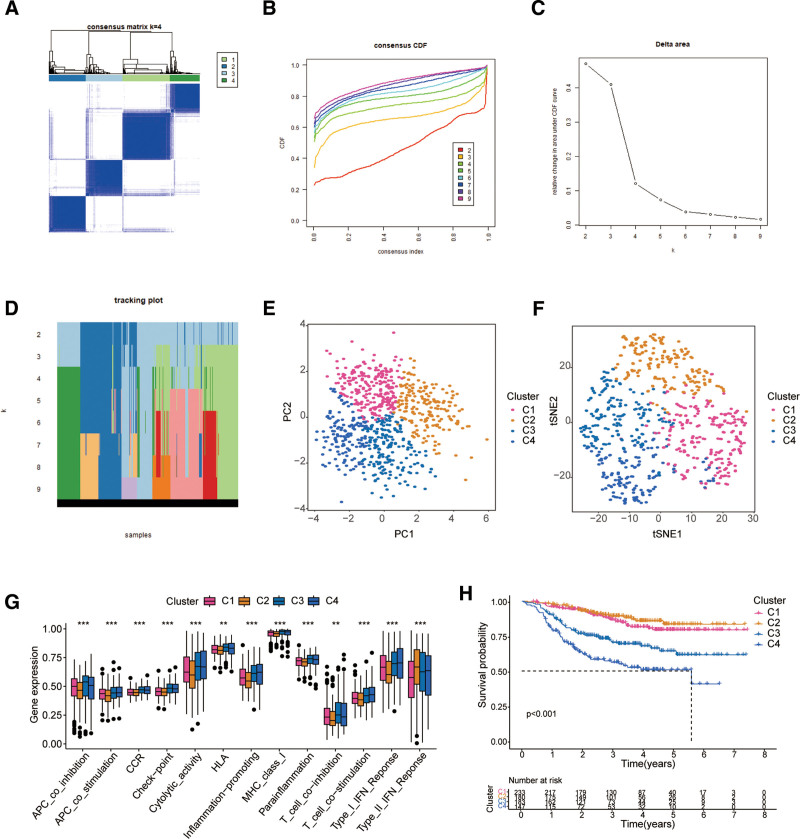
Consensus clustering of 7 OSRGs revealed 4 clusters of BC patients. (A) The Consensus matrix heatmap for K = 4. (B) Consensus clustering CDF is the relative change in the area under the CDF curve when k = 2–9. (C) Relative change in area under CDF curve for k = 2–9. (D) The tracking plot presents the clustering results when the parameter k ranges from 2 to 9. (E and F) PCA and t-SNE analysis for the 4 OSClusters. (G) ssGSEA analysis of immune-related functions among 4 OSClusters. (H) Kaplan–Meier curves was used to analyze the survival differences among 4 OSClusters. BC = breast cancer, CDF = cumulative distribution function, OSRGs = oxidative stress-related genes.

Based on the consensus cluster analysis of 7 OSRGs in the signature, we explored the relationship between oxidative stress and BC subtype, and K = 4 was the most stable and suitable choice (Fig. 8A–D). Therefore, we divided all tumor samples into 4 subgroups: cluster C1 (n = 233), cluster C2 (n = 180), cluster C3 (n = 183) and cluster C4 (n = 147). To assess the variations in immune-related functions among the 4 types of OSclusters, we employed ssGSEA analysis. PCA and t-SNE analysis showed that the molecular subtypes had good discriminative power in all 4 clusters (Fig. 8E and F). Moreover, the results showed that cluster C2 had the lowest activity for almost all types of immune-related functions, while cluster C4 showed the opposite trend, and the immune-related functional activities of cluster C1 and cluster C3 were between the 2 (Fig. 8G). Patients in Cluster C2 had a better prognosis and were less likely to relapse than those in the other 3 groups, according to a survival study between the 4 clusters; patients in Cluster C4 had the worst prognosis and were most likely to relapse (Fig. 8H).

### 4.8. The OSscore was developed to quantify individual oxidative stress patterns

**Figure 9.**
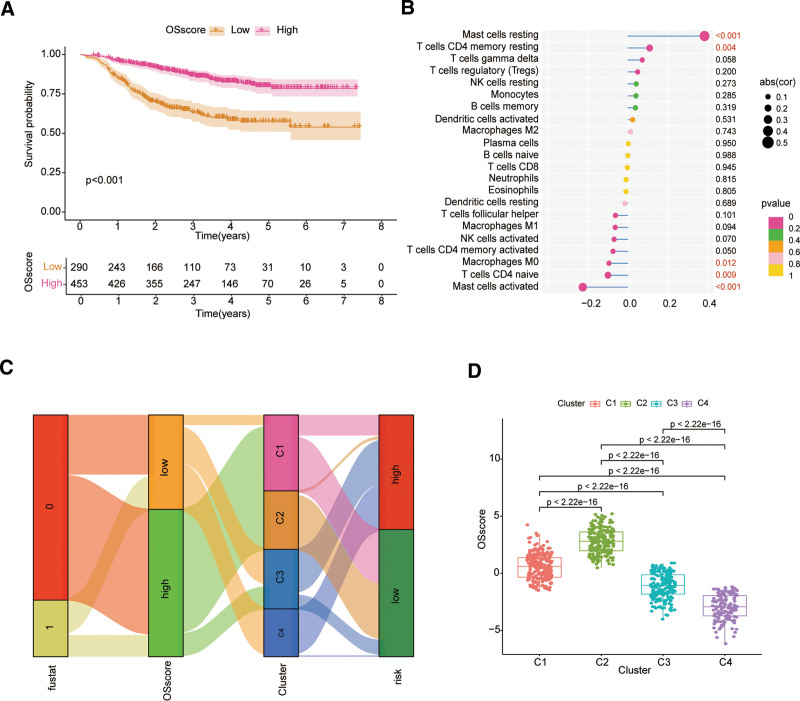
OSscore was constructed to assess oxidative stress patterns in individual patients. (A) The survival difference between high- and low-OSscore groups represented by the Kaplan–Meier curves. (B) The relationship between OSscore and immune cells. (C) The Sankey diagram of fustat, OSscore group, OSClusters group and riskScore group. (D) Differential expression of OSscore among the 4 clusters.

Given the individual heterogeneity of patients with BC, we calculated an OSscore to assess each patient’s pattern of oxidative stress based on PCA for 7 OSRGs in the signature. The scoring formula OSscore = PC1 + PC2 is used to quantify the individual oxidative stress pattern of BC patients, supporting individualized treatment. Patients with higher OSscore were more likely to relapse, according to survival analysis results (Fig. 9A). In addition, OSscore had a negative correlation with Mast cells activated, T cells CD4 naive, Macrophages M0. However,it had a favorable correlation with both Mast cells resting and T cells CD4 memory resting (Fig. 9B). According to the Kruskal–Wallis test results, the OSScore of patients in Cluster C2 was notably higher than that of the other 3 clusters, with significant differences among different clusters (Fig. 9D). The Sankey chart illustrates changes in attributes for fustat, OSscore, OScluster, and 2 risk groups. It reveals that the likelihood of recurrence in BC patients following NAC treatment increases with increasing riskScore and OSscore (Fig. 9C). All things considered, these results effectively forecast the eventual outcome of patients receiving NAC, and enrich the treatment strategy of BC patients, including not only chemotherapy, but also provide new ideas for immunotherapy.

## 5. Discussion

BC is one of the most dangerous malignant tumors, and the risk of occurrence continues to increase, which seriously threatens the lives of patients.^[15]^ While NAC improves surgical outcomes and survival for many patients, response heterogeneity and side effects necessitate better predictive tools.^[16,17]^

OS is a feature in BC, characterized by reactive oxygen species ROS imbalance, plays a dual role in tumor development.^[18]^ Although ROS contribute to genetic instability and oncogenesis,^[19,20]^ excessive ROS can also induce cell death,^[21,22]^ highlighting the context-dependent roles of OSRGs.

In our study, we identified 25 OSRGs associated with BC prognosis. Utilizing LASSO and multivariate Cox regression to eliminate duplicate components, our study developed and validated a stepwise multivariate Cox regression model with 7 OSRGs based on the GSE25066 database in order to predict the RFS of individual patients. In GSE25066 train datasets, the AUC of the ROC curve was used to examine the 1-year (0.772), 3-year (0.754), and 5-year (0.741) survival rates, combined with the KM survival curve, showing the model’s good predictive performance of patient outcomes. Specifically, a higher riskScore indicates that the patient has a higher probability of recurrence. In addition, ROC curves from 2 external independent GEO datasets confirm the reliability and accuracy of our prognostic signature and results.

The TME is the cradle of tumourgenesis and cancer progression, in which cancer cells dynamically interact with immune infiltrating cells to influence treatment outcomes.^[23,24]^ A deeper understanding of immune cell subsets within the TME could inform more effective BC therapies. We investigated patient IS, SS, and tumor purity using the ESTIMATE algorithm and computed the amount of several tumor-infiltrating immune cell types in BC using the CIBERSORT software. In the high-risk group,we found that high-risk patients exhibited increased abundance of activated CD8⁺ T cells, CD4⁺ memory T cells, and mast cells, but reduced gamma delta T cells and resting mast cells. Additionally, the findings demonstrated that the high-risk group had greater stromal and ISs, correlating with poorer prognosis. This is in line with a number of findings from earlier research.^[25–27]^ Immune checkpoint blockade has emerged as a promising therapeutic strategy that enhances antitumor immunity. Immune checkpoint inhibitors, particularly monoclonal antibodies, have shown broad clinical applications and notable successes in cancer treatment.^[28,29]^ However, their efficacy in BC remains variable.^[30]^ As a result, determining predictive indicators of how BC patients will react to various immune checkpoint therapies is essential for survival results. Our results demonstrated that the high-risk group had significantly greater expression levels of all immunological checkpoints than the low-risk group. This suggests that prognostic signature can predict the effectiveness of immunotherapy and provide guidelines for therapeutic immunotherapy regimens.

Chemotherapy remains one of the main strategies currently used to treat BC.^[31,32]^ Unfortunately, the complex heterogeneity and drug resistance of tumors can lead to a reduced response to chemotherapy drugs. Based on this phenomenon, we calculated the IC50 value, and the high- and low-risk groups responded differently to different chemotherapy drugs, so different treatment regiments for different patient groups may be more effective. In addition, riskScore is closely related to many clinicopathological parameters.

Based on the expression matrix of 7 OSRGs in the feature, we used consistent cluster analysis to identify 4 molecular clusters associated with oxidative stress. The recurrence rates of the 4 clusters were significantly different. Further investigation revealed that cluster C4 had the highest overall immune-related functional activity and the worst prognosis, whereas cluster C2 had a lower degree of immune activation and the best prognosis.

Inevitably, there are still some limitations to our study. First, with the deepening of oxidative stress research, more and more OSRGs may be identified and analyzed in the future, resulting in different analytical results and prognostic models. Secondly, although our conclusions were reached through internal and external validation of 3 cohorts in the GEO databases, there is still a need for large-scale, prospective, multicenter clinical studies in large cohorts for possible application to clinical practice. Finally, the detailed molecular mechanisms in the BC of OSRGs in the model have not been fully revealed. Further in vitro and in vivo research is necessary to investigate the links between oxidative stress and chemoresistance and to clarify the interactions between OSRGs and the tumor microenvironment.

## 6. Conclusion

To put it briefly, we constructed a novel OSRG signature and identified 4 molecular subtypes that predict RFS in BC patients and can effectively predict immune status and Prognosis in BC patients. Notably, our conclusions also offer additional clues for the accurate selection of chemotherapeutic medications in BC patients, provide a innovative perspective for immunotherapy in clinical BC, and have the potential to guide individualized cancer treatment.

## Author contributions

**Conceptualization**: Xiuping Hang.

**Data curation**: Zhongmei Shi, Xiuping Hang.

**Formal analysis**: Zhongmei Shi.

**Investigation**: Zhongmei Shi.

**Methodology**: Zhiyun Mao.

**Project administration**: Zhongmei Shi, Xiaoli Shen, Zhiyun Mao.

**Resources**: Zhongmei Shi, Xiaoli Shen, Zhiyun Mao.

**Software**: Xiaoli Shen, Zhiyun Mao.

**Validation**: Zhongmei Shi.

**Visualization**: Xiuping Hang.

**Writing – original draft**: Zhongmei Shi, Xiuping Hang.

## Supplementary Material
